# Prevention and Irony: The Challenge of Expanding Individual Behavioural Choices

**DOI:** 10.1007/s11019-026-10350-6

**Published:** 2026-04-20

**Authors:** Urban Wiesing

**Affiliations:** Institute of Ethics and History of Medicine, University of Tübingen, Tübingen, Germany

**Keywords:** Prevention, Behavioural prevention, Structural prevention, Acceleration of science, Risk, Danger, Irony, Present, Future

## Abstract

Prevention can make a significant contribution to a longer and healthier life. However, individual behavioural prevention has an influence on lifestyle with regard to how one deals with time. After all, prevention is always about making decisions in the present to change anticipated conditions in the future in a beneficial way. It is associated with challenges and an increase in responsibility. Its results are uncertain. The essay asks how one can respond to these characteristics of individual behavioural prevention. One possible response is to adopt an attitude of irony.

## Introduction

Preventive medicine enjoys an excellent reputation in the broader field of medicine. It can contribute significantly to a longer and healthier life. It should also reduce the financial burden on the healthcare system, and its role is set to become more important in the future. As research enables us to identify more and more risk factors for diseases, the scope for behavioural prevention measures is expanding. Nevertheless, prevention influences significantly how we live our life in terms of time. After all, prevention is always about making decisions in the present to change anticipated conditions in the future in a beneficial way. This text examines how this happens, the consequences and challenges it brings to the way we live our lives, and how we can react to them.

### Behavioural prevention - structural prevention

In order to assess the effects of prevention on one’s own lifestyle, it is essential to distinguish between behavioural and structural prevention. In the case of *behavioural* prevention, a person’s individual behaviour should prevent the future occurrence of an anticipated, negatively assessed health condition. In the case of *structural* prevention, on the other hand, the external conditions are to be changed for this purpose. To illustrate this, let’s consider nutrition as an example. In the case of behavioural prevention, an individual should change their eating habits by switching to a healthier diet. In the case of structural prevention, among other things, the food production industry should reduce unhealthy portion sizes, canteens should offer healthier options, taxes on sugar and alcohol should be increased and advertising for unhealthy food should be banned.

Although structural prevention is considered more successful than behavioural prevention (Philipsborn and Geffert 2021; van der Vliet et al. 2020; Loss and Leitzmann 2011), in the 20th century the focus was nevertheless set on individual behavioural prevention. The self-responsible citizen became the main object of preventive considerations, “the old, bourgeois-liberal self […] that is capable of observing itself, taking responsibility for its body and controlling its behaviour” (Sarasin 2011, p. 44, translation U.W.). The literature speaks of a development towards a “preventive self” (Lengwiler and Madarász 2014). There is a growing emphasis on individual responsibility for health, accompanied by the notion of an “entrepreneurial self” (Bröckling 2021, p. 45; see also Lupton 2013). This development goes hand in hand with other forms of individualization in modern societies. However, this preventive strategy is controversial. The WHO is criticising the inadequacy of the behavioural prevention approach to obesity, for example (WHO, 2021).

The “preventive self” is therefore not only less successful than structural prevention, but also a historically evolved concept of prevention and by no means the only possible preventive response to human sickness and mortality. Because it has a particular impact on our way of life and how we deal with time, it will be examined here. First, its conditions and structures will be explained.

## Danger versus risk: a prerequisite for prevention

An essential prerequisite for prevention is the transformation of danger into risks. A danger is a negative condition that may occur in the future and cannot be influenced. A risk, on the other hand, may also occur in the future, but can be influenced by changing present behaviour (Nettleton 2020). In other words, if there were only unavoidable dangers in the world, preventive behaviour would be pointless. As in this case, a change in behaviour in the present would not lead to a change in the future.

Due to scientific findings, more and more dangers are about to become risks. That means: more insights are being gained into how the future can be modified. Based on risk factors, we increasingly know which future events are influenced by current behaviour, and that their occurrence can be influenced by changing our behaviour in the present. This also has consequences for the understanding of illness. Scientific progress means that “illness is transformed from a danger at any time to a risk associated with lifestyle” (Luhmann 2003, p. 53).

This means that people are faced with a challenging situation. The options of prevention mean that there are several futures for a person: a future without preventive behaviour in the present, and other futures with different levels of preventive behaviour in the present. Preventive options transform an ‘uninfluenceable’ future into one that can take on different forms through action. From a medical point of view: more and more illnesses are not a matter of fate, but their future onset and severity can be influenced by behaviour in the present. This automatically results in an extension of responsibility, which may or may not be fulfilled.

## The ‘risk-ignorance-risk‘

Due to this constellation, behavioural prevention has one characteristic above all: it is intrusive. As an option, it demands reaction. Once danger has become an influenceable risk and you know about it, you can only ignore preventive options at risk. “If you ignore risks, you are already acting riskily, you are taking the *risk-ignorance-risk*.” (Fuchs 2008, p. 371, translation U.W., emphasis in original). If a person knows that certain events in the future represent an avoidable risk, then ignoring this knowledge would mean that they are taking a risk because they are ignoring the risk. Prevention thus becomes intrusive as soon as one is aware of its possibilities. This awareness is often unsolicited, as the media is full of new findings about risks and how to prevent them.

If you know about the future and the possibility of changing it, you can do something wrong without intending to, provided you do nothing. Because no response to a known risk is also a response. But is it an appropriate one? The temporal organization of life is threatened by a considerable change, impartiality is endangered.

This inevitability influences life in several ways. Prevention means being confronted with the possibility of unaddressed present day issues having an influence on the future. It calls for present-day, structured planning, in order to navigate the future. A subtle imperative to further rationalise the way we lead our lives in a preventative sense is apparent. Because knowing about a risk can still mean that something remains unaddressed. Prevention is therefore intrusive, even if you choose to ignore it, because you are aware of the preventive options. The mere possibility of prevention raises expectations that can be fulfilled or not. In this respect, prevention encompasses the possibility of failures and omissions, that the individual often has not requested.

This also creates new possibilities for moral failure (Lupton 2013). Whereas previously a certain illness was simply fate, it could prove to be controllable with increasing risk identification. The occurrence of particular illnesses could consequently be blamed on the moral failure of the sufferer. This creates further pressure to live a preventive lifestyle. A person could have prevented the illness if they had not been so lazy, so weak-willed and so inconsistent with their own body. The verdict when an illness occurs could be that the person has failed morally. It should be noted that such a moral attribution is by no means necessarily justified.

The growing possibilities mean that the dialectic of disposing and being disposed of comes to bear relentlessly in individual behavioural prevention. With the opportunity to shape the future, we have possibilities on the one hand, but on the other hand we cannot escape the possibilities and are responsible for decisions without being asked. The unsolicited burden of having to decide is the price for the new options - the agony of choice.

## Prevention in times of accelerated scientific progress

Moreover, there is no end in sight to this subtle intrusiveness! On the contrary: the options are likely to grow because of further scientific research. There is already more information on individual behavioural prevention than a person can recognise. They must therefore always make decisions based on a selection of possible information. Moreover, decisions on prevention must be made within a limited time. This is because, although preventive options are available throughout life, their number decreases towards the end of life. Always waiting for further information before starting preventive behaviour is not an effective option.

In addition, when analysing individual behavioural prevention, it should be borne in mind that medicine is in a period of acceleration. Never before has so much medical (including preventive) knowledge been gained in such a short time as at present. Consequently, a preventable present-day disease may well be treatable in the future, should it occur.

This (uncertain) hope could cause people to forego preventive measures. In addition, medical knowledge is becoming outdated faster than ever before. This also applies to preventive knowledge. Any preventive measure runs the risk of becoming outdated before it has even taken effect. Under accelerated conditions, it cannot be ruled out that new findings and new prevention options will become known before preventive behaviour takes effect or that the prevention undertaken will prove to be less effective or ineffective. Only a few preventive measures have been studied prospectively over the period in which they are intended to have an effect. In most cases, the desired effect in the longer term is derived from surrogate parameters that can be determined in the short term. Or one relies on retrospective epidemiological research, which admittedly has its own problems due to the complexity of factors that influence health. It is generally less meaningful than prospective research.

For epistemological reasons alone, it is therefore not surprising that changing risk factors and new preventive measures emerge over time. A well-known example is the reassessment of low alcohol consumption. While for a long time it was considered harmless, even beneficial to health, more recent findings have come to a different conclusion: “currently available evidence cannot indicate the existence of a threshold”. With the consequence: “Risks start from the first drop” (WHO, 2023). The example shows: Every person who wants to engage in behavioural prevention is currently confronted with more or less proven and changing preventive recommendations. Once again, it makes decisions more difficult and uncertain.

## The structures of behavioural prevention

Another aspect of individual behavioural prevention must be considered. One of the characteristic features of human behaviour in many areas of life is that the desired outcome cannot be guaranteed even if appropriate means are used. A person falls short of their own wishes, plans or options on many levels. The desired results are uncertain.

The same applies to prevention. Here, too, there are numerous possibilities for failure or success, which can be systematised. If successful prevention requires (1) the selection of a goal that a person wants to strive for; (2) measures with which the goal can actually, not allegedly, be achieved; (3) which are also practiced; and (4) the goal is then also achieved (which cannot be guaranteed), then success and failure can be structured according to these conditions.

You can fail at any of these points. (1) the intended goal was not appropriate for a person’s lifestyle. A person can be mistaken in their preventive goals, or the preventive goal is unrealistic. Not all aspirations have a realistic chance of being realised. (2) the wrong means were used, (3) the means were not practiced, and (4) the desired goal was not achieved despite correct practise of adequate means. A person’s health is the result of a complex interplay of numerous factors that can always be influenced to a certain extent by prevention, but never completely. Even measures that have been successfully implemented cannot guarantee with certainty that diseases will be prevented. The occurrence of a disease does not automatically mean that no preventive measures were taken nor means a long and healthy life automatically that preventive measures were taken. Only statistically correlations can be demonstrated.

In addition to all the imponderables, there is also a risk that a simple coincidence, e.g. a traffic accident, will thwart the calculation of a consistently preventive lifestyle. Before the predicted gain in lifespan could be achieved through prevention, other contingencies have put an end to life. In short, the hoped-for quantitative and qualitative gain in lifespan through behavioural prevention cannot be expected with certainty, but only with a certain probability.

It should not be withheld that prevention not only can fail but likewise can succeed and does succeed for many people. It is highly likely that they can expect to improve their lifespan and quality of life through behavioural prevention. Inverting the previous statements about failure reveals the structures of success: anyone who (1) wants a long and healthy life and is at peace with themselves, (2) chooses the right methods, (3) practices them and (4) therefore extends their healthy lifespan in quantitative and qualitative terms, can enjoy successful prevention. A person who enjoys a healthy diet and exercise, for example, will benefit from this lifestyle in the present and probably also in the future. They strive to achieve their own goals with pleasure and identification with themselves. A willingly practiced intensification of the present experience of time through preventive behaviour, e.g. yoga, leads to extensional time gain. Even if the goal of prolonging life is not achieved due to an unfortunate coincidence, the time spent for preventive measures beforehand would still be a successful one, as it has already been experienced positively.

It should be added that, unlike structural prevention, individual behavioural prevention is subject to a social bias. It is primarily the wealthy who benefit from it, rather than poorer people (Graham 2007).

## Experience of time: behavioural prevention and the temporal organization of life

In this respect, individual behavioural prevention always has two aspects in relation to experience of time. It changes the experience of the present time through practiced, preventive actions and aims to gain additional time in the future through present practiced, preventive actions. The relationship between prevention and a good life is therefore twofold. On the one hand, prevention can contribute to a good life by creating more healthy time in the future. On the other hand, prevention can also contribute to a good life in the present because preventive measures immediately benefit those who enjoy engaging in them. For example, this applies to people who enjoy sports and eating healthily, and who don’t find it difficult to give up alcohol and tobacco.

Nevertheless, the opposite is also possible. For many people, preventive behaviour is not a pleasure, but an unpleasant activity, a burden. For these people, a paradox looms: individual behavioural prevention could become counterproductive. If preventive measures are estimated as a burden, more negatively valued, burdensome time could be spent on prolonging life than life is prolonged. Depending on how one evaluates the time spent on preventive behaviour, the balance could be negative.

Another aspect regarding time and prevention must be addressed. By focusing on the future and shaping it through current measures, lifestyle is more oriented towards the future. Behavioural prevention supports a phenomenon that Hermann Lübbe has termed “present shrinkage” (org. “Gegenwartsschrumpfung”) (Lübbe 1997; translation U.W.). The future orientation of prevention brings the future closer and leads to a shortened stay in the present. The significance of the present shrinks; it is to be shaped towards the future, in extreme cases it becomes a “bourgeoisly slowed down, highly insured waiting for death, which must be postponed as long as possible” (Fuchs 2008, p. 374, translation U.W.).

In other words: Worrying about the future can affect the present and presentness. It is about the future that should not happen, with the risk that the present no longer takes place in relation to the present, but in relation to the future. Preventing future illness runs the risk of missing out on the present. Futurity can prevent presentness.

A distinction mentioned at the beginning should be made here once again: structural and behavioural prevention differ considerably in relation to an individual’s perspective on time. Structural prevention, such as reducing the concentration of pollutants at work, has hardly any influence on a person’s temporal structures and is therefore quite different from lifestyle change for preventive reasons (behavioural prevention). The same applies to advertising bans or increased taxes on unhealthy eating, which influence behaviour indirectly. Behavioural prevention, on the other hand, directly influences the timing of human life. People change their behaviour in the present for preventive reasons in order to benefit from it in the future. It is always about “influencing future presents through decisions in the here and now” (Leanza 2011; translation U.W.). In addition, behavioural prevention always requires individual motivation and implementation of actions, whereas structural prevention is usually organised collectively. As a result, it has less influence on how people shape their lives on their own initiative.

## What to do?

In summary, the following situation arises regarding individual behavioural prevention: more and more dangers are becoming risks due to scientific findings. Without being asked, people have increased opportunities to change their behaviour in the present in order to gain a longer and healthier life in the future. Diseases are increasingly less a matter of fate than of preventive behaviour that is not practiced. However, gains are not certain; failure is possible for many reasons. It is only realistic that the gain will not be 100%. Something planned might not be realised and something realised might not lead to the desired success, sometimes more, sometimes less. Even with such preventive actions, life can still unfold in ways where a person falls short of their own wishes, plans or options on many levels.

For many people, changing their behaviour in the present already means a gain with the prospect of a longer and healthier future. For many others, however, it means effort and stress, discipline and burden. For these individuals, behavioural prevention can lead to a paradox: the unpleasant, stressful time spent on prevention is greater than the time gained through prevention. However, not dealing with the unsolicited possibility of prevention at all leads to the risk of risk ignorance. In this respect, it is impossible to escape the new, unsolicited options. This basic structure of individual behavioural prevention can therefore lead to disappointment and stressful feelings of guilt due to alleged moral failure.

## An attitude of irony as a response

How should we react to the characteristics of individual behavioural prevention and how should we deal with it? One can face unexpected and complex challenges in life with the right attitude. Philosophers have known this since antiquity. The question then arises: What attitude is recommended in view of the characteristics of individual behavioural prevention?

It has proven to be wise to adopt an ironic attitude towards the unquestioned and unavoidable possibility of failure - and not only in this respect.^1^ What would that mean? First of all, irony means detaching oneself, independently distancing oneself from the immediate intrusiveness of what is happening. When a person is confronted with the structures of individual behavioural prevention without being asked, it can prove to be a relief to keep a distance from the overwhelming immediacy and to face it with serenity. Facing the possibility of success or failure with irony makes it less threatening. The permanent confrontation in the present with the certainty of uncertain future success is easier to bear with irony.

According to Richard Rorty, ironists are “never quite able to take themselves seriously” (Rorty 2012, p. 73). And they would suggest that life should not always be interpreted “as an emergency” (Bozzaro 2014, p. 233, translation U.W.). Ironists are likely to counter such an assessment with self-irony and try not to take themselves too seriously. In addition, irony requires a joyful renunciation of a final assurance of what is the only right thing to do. Because - according to ironists - there is no such thing in life as there isn’t in behavioural prevention.

Ironists would view the individual behaviour prevention less from a moral standpoint and more from the perspective of taste. The “generic task of the ironist” is “to create the taste by which he will be judged. But the judge the ironist has in mind is himself“ (Rorty 2012, p. 97). This attitude is helpful for individual behavioural prevention. For ironists, the preventive shaping of their own lives does not require them to be bound to a higher authority, which sets the standards for prevention and carries out a retrospective evaluation. All of this remains with the ironist himself, who does this with a certain attitude of distance.

Prevention endeavours to prolong life. For an ironist the following applies: “If he had been alive or sane longer, there would have been more material to be rearranged, and thus different redescriptions, but there would never have been the right description” (Rorty 2012, p. 99). This statement applies to prevention in two respects: prevention should prolong life, and with an ironic attitude this also allows further “redescriptions” of one’s own life. This attitude reduces the threat while looking at one’s own life. Simply because the objectives of prevention lose their immutability and exclusivity with an ironic attitude, there can be a re-evaluation of changing convictions, the unplanned and errors. If you accept the uncertainties of life and see possible change as an opportunity for reorientation and redefinition, you can look back on your life with more serenity. This provides relief in the face of the unquestioned imposition of the ‘risk-ignorance-risk’ and allows failure to be seen in a milder light. The very likely failures do not automatically become tragic. This promotes self-protection.

As with other areas of life, the respective efforts at individual behavioural prevention can also be viewed from different perspectives, with no single perspective having exclusive significance (Randall 2013b). An ironist may see his attempts at individual behavioural prevention as a selection of possible responses to a confluence of contingent social, technical, and other circumstances. His preventive behaviour is to be understood as a possible, but not obligatory arrangement in the face of the most diverse contingencies. He lives by chance and without being asked in a time in which increasing knowledge of risk factors enables increasing preventive options.

Moreover, reactions to new constellations are always something open-ended, a process that allows for new aspects and evaluations. For this reason, irony is a good prerequisite for redescribing, reinventing and realigning oneself. This creates more opportunities to find one’s own appropriate lifestyle. In view of the rapid changes in our society and the technological possibilities, this can also be very helpful in individual behavioural prevention. After all, in the age of accelerated science, we must expect to be confronted with new findings and to have to revise old certainties. Prevention decisions made in the past do not always have to be proven wise. A clever prevention strategy should not rule out changing, adapted reactions and should not necessarily interpret a new perspective on life as failure. After all, every person faces the challenge of finding their own degree of consistency and flexibility in prevention. In addition, irony makes it possible to allow ambiguities in one’s own self-image. Several perspectives on one’s own preventive biography can be juxtaposed.

For Mariacarla Gadebusch Bondio, prevention is a “paradigmatic expression of a competition with time” (Gadebusch Bondio 2017, p. 37, translation U.W.). For ironists, this does not necessarily have to be the case. They would like to give the competition a different character. For them, it should not only have combative aspects, such as the classification as winner or loser, but also opportunities for new self-descriptions and new self-creations. Instead of “competition”, the ironist recommends an assessment as a game. Irony defuses competition, allows playfulness in the preventive lifestyle, a realization and interpretation of preventive actions with imagination and self-distance.

Moreover, irony can promote solidarity (Rorty 2012). Other people are also faced with an increasing responsibility for complex structures of individual behavioural prevention, despite not being asked to do so, and will not only be successful in their preventive efforts. This connects! Irony provides mortals with an opportunity to collectively deal with the pitfalls of the *conditio humana* in the age of technologically influenced life.

Whilst this article advocates irony as an element of a response to the characteristics of individual behavioural prevention, this is, on the one hand, a novel approach (Wiesing 2023). However, on the other hand, it forms part of a tradition of responses to challenging situations. Adopting certain attitudes to address complex challenges has been a philosophical response since antiquity. This is also a subject of current debates, including in relation to irony.^2^ In this respect, a long philosophical tradition is being applied to the new challenges of individual behavioural prevention. The precautionary, future-oriented conduct of life is subject to the same considerations of wisdom as other parts of life. An attitude of irony can be an element of this wisdom.

## Footnotes

1 Interestingly, the contemporary discussion of temporality, medicine and the good life has rarely addressed irony as an element of an appropriate response, with the exception of Randall 2013a, b in relation to ageing. A search on pubmed (14.4.2025) did not yield any further results on the topic of irony as an appropriate attitude in prevention.

2 Thomas Nagel 1971 recommends irony as an attitude in the face of the absurdity of human existence: „If sub specie aeternitatis there is no reason to believe that anything matters, then that doesn’t matter either, and we can approach our absurd lives with irony instead of heroism or despair.” (p. 727).
